# Does Aerobic plus Machine-Assisted Resistance Training Improve Vascular Function in Type 2 Diabetes? A Systematic Review and Meta-Analysis of Randomized Controlled Trials with Trial Sequential Analysis

**DOI:** 10.3390/jcm11154257

**Published:** 2022-07-22

**Authors:** Xianshan Guo, Shizhe Guo, Hongmei Zhang, Zhen Li

**Affiliations:** 1 Department of Endocrinology, Xinxiang Central Hospital/The Fourth Clinical College of Xinxiang Medical University, Xinxiang 453000, China; gxs52@163.com; 2 Department of Endocrinology, Yangpu Hospital, School of Medicine, Tongji University, No. 450 Tengyue Road, Yangpu District, Shanghai 200090, China; guoshizhe@126.com; 3 Yangpu Mental Health Center, No. 585 Jungong Road, Yangpu District, Shanghai 900093, China; 4 Department of General Surgery, Yangpu Hospital, School of Medicine, Tongji University, No. 450 Tengyue Road, Yangpu District, Shanghai 200090, China

**Keywords:** diabetes mellitus, aerobic training, resistance training, vascular function, meta-analysis

## Abstract

Type 2 diabetes mellitus (T2DM) is a chronic disease characterized by hyperglycemia, insulin resistance, and pancreatic B cell dysfunction. Hyperglycemia can cause several complications, including nephrological, neurological, ophthalmological, and vascular complications. Many modalities, such as medication, physical therapies, and exercise, are developed against vascular disorders. Among all exercise forms, aerobic plus machine-assisted resistance training is widely applied. However, whether this intervention can significantly improve vascular conditions remains controversial. In this study, an electronic search was processed for the Pubmed, Embase, and Cochrane libraries for randomized controlled trials (RCTs) comparing the efficacy of aerobic plus machine-assisted resistance training with no exercise (control) on patients with T2DM. Pulse wave velocity (PWV), the index of arterial stiffness, was chosen as primary outcome. The reliability of the pooled outcome was tested by trial sequential analysis (TSA). Secondary outcomes included systolic blood pressure (SBP) and hemoglobin A1c (HbA1c). Finally, five RCTs with a total of 328 patients were included. Compared with control, aerobic plus machine-assisted resistance training failed to provide significant improvement on PWV (MD −0.54 m/s, 95% CI [−1.69, 0.60], *p* = 0.35). On the other hand, TSA indicated that this results till needs more verifications. Additionally, this training protocol did not significantly decrease SBP (MD −1.05 mmHg, 95% CI [−3.71, 1.61], *p* = 0.44), but significantly reduced the level of HbA1c (MD −0.55%, 95% CI [−0.88, −0.22], *p* = 0.001). In conclusion, this meta-analysis failed to detect a direct benefit of aerobic plus machine-assisted resistance training on vascular condition in T2DM population. Yet the improvement in HbA1c implied a potential of this training method in mitigating vascular damage. More studies are needed to verify the benefit.

## 1. Introduction

The prevalence of Type 2 Diabetes Mellitus (T2DM) is increasing rapidly worldwide [1]. Characterized by hyperglycemia, insulin resistance, and pancreatic B cell dysfunction, T2DM leads to several severe complications, including but not limited to neuropathy, nephrology, and macrovascular disorders such as cardiovascular diseases [2,3]. Compared to healthy population, the risk of cardiovascular events has a twofold increase [4]. Cardiovascular event is also the leading cause of mortality in patients with T2DM [5]. Therefore, maintaining the function of vessels or retaining the damage of vessels is of vital importance.

Lifestyle modifications, such as diet control, nutrients supply, body weight regulation, and sports exercise, are well-acknowledged to improve the prognosis of T2DM [4,5,6,7]. Sports exercise can be divided into aerobic training, resistance training, or combination. As an easily accessible pattern, aerobic training helps control blood pressure, systemic inflammation, and glycemic level, et al. [8]. Way et al. found aerobic training could improve smooth muscle function, but the improvement of vascular stiffness was still questioned [9]. Alternatively, resistance training can change body composition by increasing the mass of muscle, which is important in controlling blood glucose and ameliorating insulin resistance [10]. When combined with aerobic training, this strategy may improve vascular function in healthy individuals [11]. In 2014, Li et al. found that combined aerobic and resistance training was beneficial for decreasing arterial stiffness in population with or without hypertension [12]. These findings provide the rationality of this combined training for patients with T2DM.

In recent years, clinical trials have been launched to test whether aerobic plus resistance training is beneficial to the vascular complications of T2DM. To better understand where we are now, a systematic review and meta-analysis is organized to verify the effect of aerobic plus machine-assisted resistance training on the vascular condition in patients with T2DM.

## 2. Materials and Methods

This systematic review was organized according to the PRISMA (Preferred Reporting Items for Systematic Reviews and Meta-analyses) checklist [13].

### 2.1. Search Strategy

On January 2022, the first two authors independently searched on Pubmed, Embase, and Cochrane library. Reference lists of previously published systematic reviews were also reviewed related researches. Key words used were, random *, (vessel * or cardiovascular or vascular), diabet *[title/abstract], and (exercise or training).

### 2.2. Study Selection

Studies focusing on the comparison of resistance plus aerobic training and no or sham training for improvement of the vascular function in T2DM population were included. The inclusion criteria were (1) randomized controlled trials (RCTs), (2) an intervention consisted of a combination of machine-assisted resistance training and aerobic training, and (3) intervention duration for at least four weeks [14]. The exclusion criteria were (1) non-randomized control trials, (2) animal studies, and (3) RCTs in which the intervention group did not have a combined training protocol, (4) combined protocol in which resistance training protocol was unclear or not machine-assisted, and (5) non-randomized clinical trials, case reports, reference abstracts, or reviews. The first two authors independently screened titles and abstracts of all searched items based on the criteria above. Once the information to make a decision was insufficient, full-text would be retrieved for further judgment. In case of debate, the senior author would decide whether to include the research.

### 2.3. Data Extraction

The same authors independently extracted data from eligible studies including name of first author, published year, inclusion and exclusion criteria, number of patients included, training protocols, and items of measurements, as well as conclusions. The difference of changes of central pulse wave velocity (PWV) between two groups was selected as the primary outcome, since PWV is not only an indicator arterial stiffness but also an independent predictor of cardiovascular risk [15]. Secondary outcomes included the difference of changes of systolic blood pressure (SBP) and hemoglobin A1c (HbA1c) between two group. The former reflects vasculature plasticity [16], while the latter is used for evaluating blood-glucose control over a period of time and to predict the occurrence of long-term complications due to diabetes [17].

### 2.4. Data Analysis

The random-effects model was applied for each comparison since patient conditions, exercise duration and modes, as well as other factors were inconsistent across RCTs. Difference in primary and secondary outcomes were measured by mean difference (MD) and 95% confidence interval (CI). For researches in which the standard deviation (SD) of pre-intervention and post-intervention difference was not reported, a correlation of 0.5 was used for dispersion estimation [18]. For researches with multiple eligible intervention groups, the control group was split equally based on the number of intervention groups, and two or more comparison pairs were input [19]. Heterogeneity was assessed by Q statistic and I^2^ statistic. I^2^ statistic larger than 50% were considered to have significant heterogeneity [20]. When significant heterogeneity was noticed regarding primary outcome, sensitivity analysis was conducted. One study was omitted in each turn to locate the potential source of heterogeneity. Since the number of RCTs included did not reach ten, publication bias was not detected [21]. Two-tail *p* value < 0.05 was considered statistically significant. Analyses were performed using Review Manager, Version 5.3 (The Nordic Cochrane Centre, The Cochrane Collaboration; Copenhagen, Denmark).

### 2.5. Quality Assessment

The Cochrane’s risk of bias tool was used by the first two reviewers independently assess the quality of included studies [22]. Value of low, unclear or high risk of bias was assigned to the following items: random sequence generation, allocation concealment, blinding of participants and personnel, blinding of outcome assessment, incomplete outcome data, selective reporting and other bias. Disagreement was solved by discussion. The degree of inter-reviewer agreement was measured by κ value. A κ from 0.40 to 0.59 was regarded as fair, 0.60 to 0.74 as good, 0.75 or more as excellent [22].

The quality of evidence for primary outcome was rated by the Grading of Recommendations Assessment, Development, and Evaluation (GRADE) approach. The level of evidence was entitled as high, moderate, low, or very low, according to five domains: high risk of bias, imprecision, indirectness, heterogeneity, and publication bias [23,24,25,26,27]. Considering the limited number of studies included, publication bias could not be assessed. Instead, evidence was downgraded when heterogeneity exceeded 40% [25].

### 2.6. Trial Sequential Analysis

Given sparse data and repeated significance testing, the risk of type I error might be elevated by cumulative meta-analyses [28,29,30,31]. To control this potential risk, trial sequential analysis (TSA) was launched (TSA software version 0.9 Beta; Copenhagen Trial Unit, Copenhagen, Denmark) for all measurements by empirical method for the estimation of the required information size. The diversity-adjusted required information size (DIS) and the eventual breach of the cumulative Z-curve of relevant trial sequential monitoring boundaries was obtained to calculate the required information size together with a threshold for a statistically significant treatment effect [32]. An overall 5% risk of a type I error was maintained with a power of 80% [32].

## 3. Results

**Figure 1:**
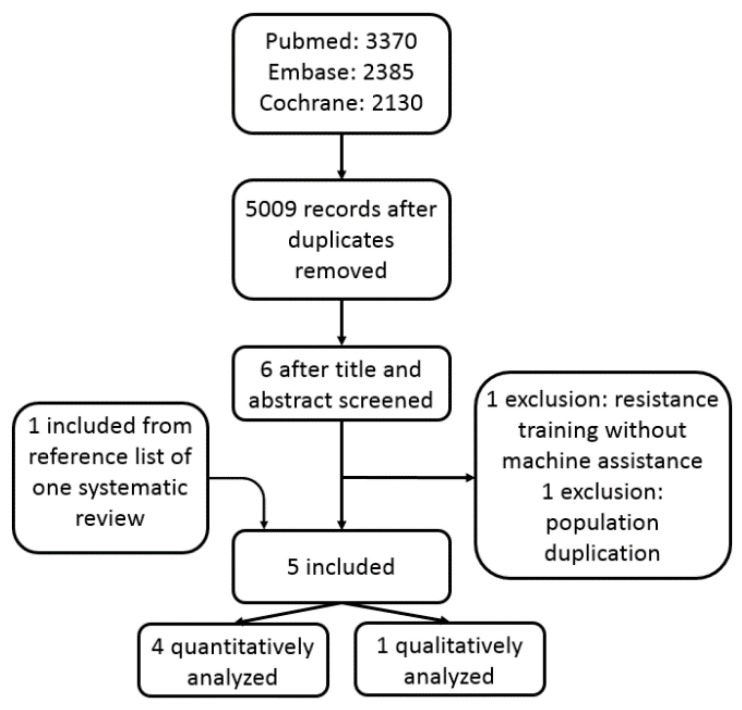
The flowchart of study inclusion.

A total of 4838 titles were identified after electronic screening in three databases. After reading titles and abstracts, the full-text of seven titles were retrieved for further exclusion. The resistance training in the trial reported by Okada et al. [33] was not machine-assisted, therefore was excluded. Two studies shared the same patient cohort, so the latter one, which was a secondary analysis of the original population, was excluded [34]. One eligible study [35] was identified from a systematic review [14], and was included (Figure 1).

Five RCTs with a total of 328 patients were included, of which four RCTs were quantitatively analyzed [35,36,37,38], while one was descriptively analyzed [39]. Basic characteristics of these studies were listed in Table 1. These researches were published from 2001 to 2019. A total of 176 patients with T2DM were allocated to aerobic plus resistance training group. The age range of the patients included in the systematic review was from about 40 to over 60 years old. In one study, only male participants were enrolled [35]. Detailed intervention, follow-up duration, and conclusions were listed in Table 2. The aerobic training consisted of cycle ergometry, walking, or treadmill et al., while resistance training focused mainly on trunk and extremities on machines. All training processes use a heart rate detector to determine the quality and quantity of exercise. Started from the beginning of exercise, two RCTs had a follow-up of 52 weeks [35,38], two had a follow-up of 26 weeks [36,37], and one had 16 weeks [39].

**Figure 2:**
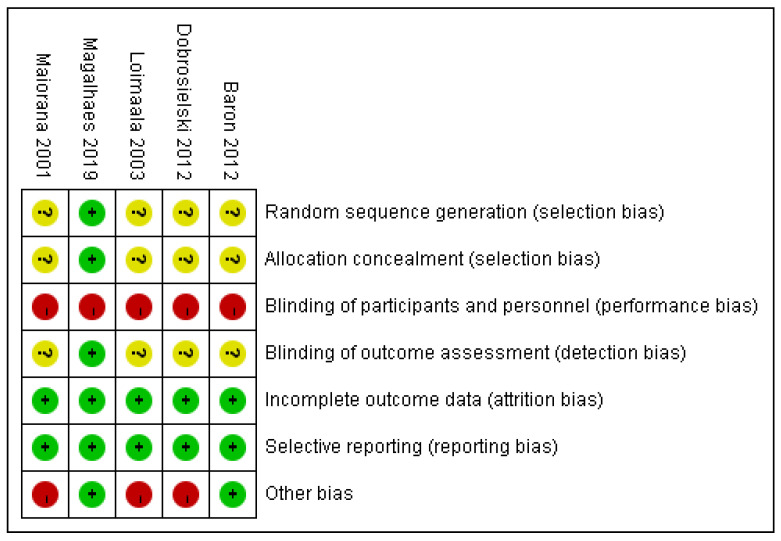
Risk of bias of each included study [35,36,37,38,39]. +: low risk; -: high risk; ?: unclear risk.

Risk of bias of included studies was shown in Figure 2. Most of the studies did not mention the detail of randomization or allocation concealment. Considering the nature of exercise process, it was impossible to keep patients blinded to interventions. No studies had incomplete outcome data or selective reporting. Regarding other biases, two of the five studies had sample size calculation prior to patient enrollment, and therefore was ranked as low risk [36,38]. The κ value was 0.82, indicating an excellent consistency between two reviewers.

### 3.1. Primary Outcome

**Figure 3:**
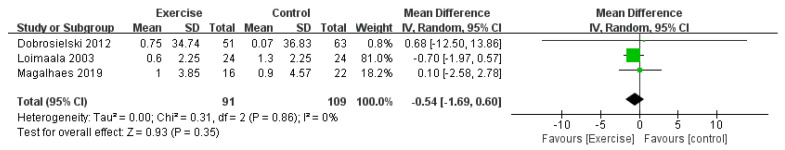
The pooled result of the difference of changes in pulse wave velocity between two groups [35,37,38].

**Figure 4:**
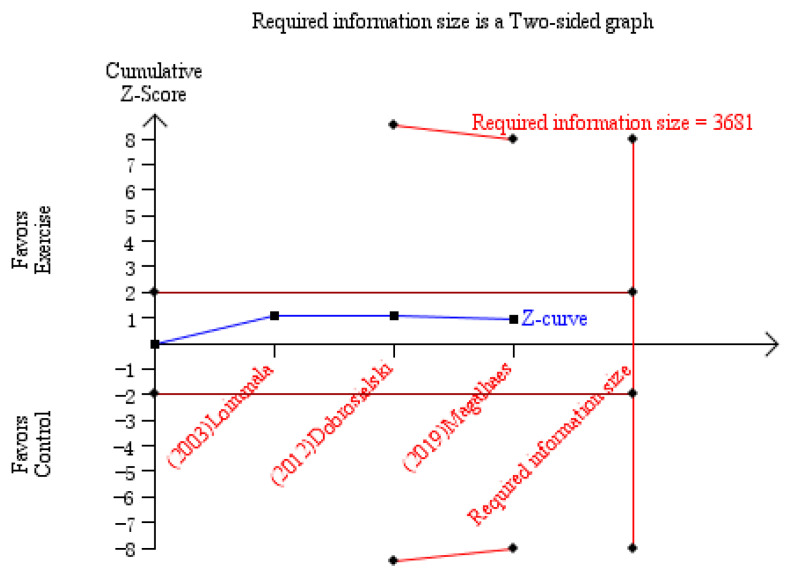
The result of TSA for PWV. TSA showed that the pooled results did not (z-curve, blue curve) crossed the conventional boundary of benefit (brown line) or the trial sequential monitoring boundary for benefit (upper red line), and did not reach the required sample size based on TSA (*n* = 3681) [35,37,38].

Compared with control, aerobic plus resistance training did not significantly improve PWV of patients with T2DM (MD −0.54 m/s, 95% CI [−1.69, 0.60], *p* = 0.35, three studies included [35,37,38]). The heterogeneity was not remarkable (I^2^ = 0%, *p* = 0.86) (Figure 3). Considering that study design might introduce bias and some data were calculated based on estimation, the level of evidence was low. However, this insignificance was not supported by TSA, which indicated the current outcome might be a result of limited sample size (Figure 4).

### 3.2. Secondary Outcomes

**Figure 5:**
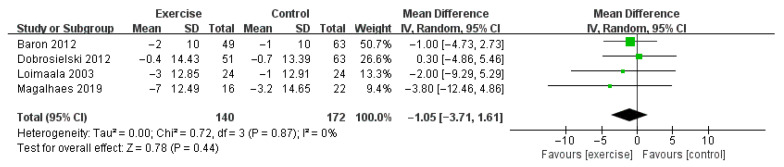
The pooled result of the difference of changes in systolic blood pressure between two groups [35,36,37,38].

Compared with control, aerobic plus resistance training did not significantly improve SBP of patients with T2DM (MD −1.05 mmHg, 95% CI [−3.71, 1.61], *p* = 0.44, four studies included [35,36,37,38]). The heterogeneity was not significant (I^2^ = 0%, *p* = 0.87) (Figure 5).

**Figure 6:**
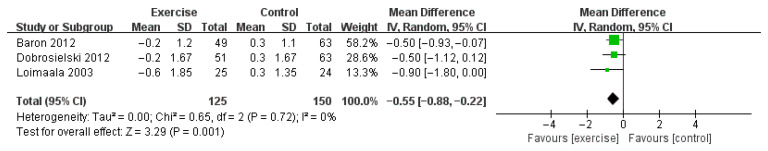
The pooled result of the difference of changes in hemoglobin A1c between two groups [35,36,37].

On the other hand, aerobic plus resistance training significantly decreasedHbA1c of patients with T2DM (MD −0.55%, 95% CI [−0.88, −0.22], *p* = 0.001, three studies included [34,36,37]). The heterogeneity was insignificant (I^2^ = 0%, *p* = 0.72) (Figure 6). This outcome was in consistent with the records of Maiorana et al. [39] that, at the final follow-up, the level of HbA1c was 7.9 ± 0.3% in patients with exercise, significantly lower than those without exercise (8.5 ± 0.4%).

## 4. Discussion

Numerous meta-analyses have discussed the benefit of various types of exercise training on T2DM in glycemic control, psychosocial performance, the level of inflammatory cytokines, et al. [40,41,42]. In the current study, we primarily focused on the effect of aerobic plus machine-assisted resistance training on vascular function in patients with T2DM. Based on the data available, we failed to detect a statistical significance of this combined training method for vascular condition, as indicated by PWV or SBP. On the other hand, TSA suggested that this insignificant difference might be attributed to a relatively small sample size. Moreover, aerobic plus machine-assisted resistance training significantly reduced the level of HbA1c, which is associated with cardiovascular risk. Therefore, it is currently not appropriate to negate the benefit of this training method on vascular health in T2DM population.

Resistance training can be either machine-assisted [38,39], elastic band-assisted [43], or even free weight [44]. Some studies used elastic bands in resistance training section, but did not notice an improvement in flow-mediated dilation or endothelium-independent vasodilation in T2DM population [43,45]. On the other hand, aerobic plus free weight-based resistance training could significantly decrease carotid intima-media thickness and arterial stiffness [44,46]. Considering that the uncertainty in body weight or the elasticity of bands may act as confounders, we focused specifically on machine-assisted resistance training.

As the widely used structural and functional index for measuring arterial stiffness [47], PWV is usually faster in T2DM population [48], indicating vascular stiffening and high cardiovascular risk [49]. Surprisingly, the present data did not support the application of this exercise protocol for improving vascular condition in T2DM population. This may be explained by two factors. First, as aforementioned, those receiving free weight exercise have improved vascular condition, so one can speculate that different resistance training protocol may yield different outcomes. Second, by conducting TSA, we noted that the required sample size was not reached, therefore more clinical trials are still needed.

Next, we compared the change in hemodynamic index, SBP. As an reflection of the plasticity in vasculature [16], SBP is always higher in stiffened vessel [50]. We found that the change of SBP following aerobic plus resistance training was comparable to that in control group, indicating that this training protocol may not be able to improve vascular function in T2DM population.

However, in agreement with a recent meta-analysis [42], we noticed a significant decrease of HbA1c in T2DM population with exercise, implying that aerobic plus machine-assisted resistance training could help control blood glucose. In hyperglycemia-induced complications of T2DM, especially vascular dysfunction, oxidative stress plays a pivotal role [51]. Oxidative damage caused by excessive reactive oxygen species can lead to endothelial damage via several signaling pathways, aggravating vascular stiffness and impairing vasorelaxation [52,53]. To prevent or retard the progression of complications, long-term control of blood glucose is of vital importance [54], the benefit of aerobic plus machine-assisted resistance training on blood glucose control was an indirect evidence that this exercise protocol could be meaningful for controlling vascular complications. This was in accordance with previous researches that high HbA1c was associated vascular risk and could be predictive of vascular events [55,56].

Previously, several systematic reviews and meta-analyses studied the influence of exercise on vascular function in T2DM. By measuring brachial artery flow-mediated dilation, Lee found that exercise as a whole, regardless of the pattern, significantly improved vascular endothelial function [57]. Dos et al. noticed that aerobic plus resistance training could improve vascular function in T2DM [14]. However, some of the included studies were not RCTs, which may act as the origin of divergence compared with ours.

The current findings should be interpreted with caution. First, some data we input were based on estimation, which might introduce impreciseness. The sample size was also not statistically sufficient, as indicated by TSA. In addition, the relatively short follow-up duration may contribute to the insignificant difference of PWV or SBP. Contrarily, we found that HbA1c was improved by training. Since HbA1c is related to better glucose metabolism, which indicates greater redox balance, one can expect a better vascular system [58]. Next, the evaluation of vascular condition should be multi-dimensional. A comprehensive understanding of vascular status in T2DM patients with exercise can be conducted in the future. Thirdly, albeit an exact protocol of exercise in each study, confounders were unavoidable. The age ranges from 40 to over 60, the follow-up duration ranges from 16 to 52 weeks, and even one study only recruited male patients. To reveal the effect of aerobic plus resistance training on vascular health, a longer follow-up period in a group of patients with closer age range is necessary. Finally, HbA1c does not indicate the variation of the glycemic profile, which is also a risk factor for cardiovascular events in T2DM population [59]. The effect of exercise on glycemic variability can be detected in further trials.

In conclusion, the outcome of the current meta-analysis was not supportive of the benefit of aerobic plus machine-assisted resistance training on vascular condition in T2DM population. However, this finding could be a result of small sample size. Considering that there was a significant improvement of HbA1c after training, this method may still have the potential of maintaining vascular health. More studies with longer follow-up duration are required to verify this potential.

## Figures and Tables

**Table 1:** Basic characteristics of included studies.

Number	Title	Authors	Year of Publication	Participants		Age	
				Exercise	Control	Exercise	Control
1	The effect of combined aerobic and resistance exercise training on vascular function in type 2 diabetes	Maiorana et al.	2001	6	16	52 ± 8 as a whole	
2	Exercise training improves baroreflex sensitivity in type 2 diabetes	Loimaala et al.	2003	24	25	53.6 ± 6.2	54 ± 5
3	A randomized trial of exercise for blood pressure reduction in type 2 diabetes: Effect on flow-mediated dilation and circulating biomarkers of endothelial function	Baron et al.	2012	49	63	58 ± 5	56 ± 6
4	Effect of exercise on blood pressure in type 2 diabetes: a randomized controlled trial	Dobrosielski et al.	2012	70	70	57 ± 6	56 ± 6
5	Effects of combined training with different intensities on vascular health in patients with type 2 diabetes: a 1-year randomized controlled trial	Magalhaes et al.	2019	28	27	59.7 ± 8.3	59.0 ± 6.5

**Table 2:** Measurements, exercise protocols, follow-up duration, and findings.

Number	Analyzed Measurements	Aerobic Training Protocol	Resistance Training Protocol	Follow-Up	Conclusion
1	I: Changes in forearm blood flow II: Endothelium-dependent, flow-mediated dilation of brachial artery III: Endothelium-independent glyceryl trinitrate-mediated dilation of brachial artery	A combination of cycle ergometry and treadmill walking maintained at 70% to 85% of peak heart rate.	Leg press, hip and shoulder extension, pectoral exercises, seated abdominal flexion and dual leg flexion on weight-stack machines, with an intensity 55% to 65% of pretraining maximum voluntary contraction.	16 weeks	This study supports the value of an exercise program in the management of type 2 diabetes.
2	I: Systolic blood pressure II: Pulse wave velocity III: Systemic vascular resistance indexIV: HbA1c	Jog or walk twice a week at a heart rate level of 65–75% maximal oxygen consumption	Eight sessions for large muscle groups from the trunk and upper and lower extremities with three sets of 10–12 repetitions at 70–80% maximum voluntary contraction.	52 weeks	No significant changes in systemic hemodynamics were observed.
3	I: Blood pressure II: HbA1c III: Lipids IV: Endothelial biomarkers V: BMI, body and visceral fat VI: Endothelium-dependent, flow-mediated dilation of brachial artery	A 10–15 min warm-up, 45 min of aerobic exercise at a target heart rate between 60 and 90% of maximum heart rate, and a cool down.	Weight training exercises (latissimus dorsi pull down, leg extension, leg curl, bench press, leg press, shoulder press, and seated mid-rowing) for 2 sets of 12–15 repetitions at 50% of 1-repetition maximum.	26 weeks	There were no changes in endothelium-dependent flow-mediated dilation or circulating endothelial biomarkers.
4	I: Resting systolic and diastolic blood pressure II: Diabetes status III: Pulse-wave velocity IV: Body composition and fitness	45 min for treadmill, stationary cycle, or stairstepper with a target range of 60% to 90% of maximum heart rate.	Two sets of 7 exercises at 10 to 15 repetitions per exercise at 50% of 1-repetition maximum on a multistation machine	26 weeks	The lack of change in arterial stiffness suggests a resistance to exercise-induced blood pressure reduction in persons with T2DM.
5	I: Systolic and diastolic blood pressure II: HbA1c III: Conduit artery intima-media thicknessIV: Carotid blood pressure V: pulse wave velocity VI: Physical activity and fitness	Continuous cycling with 40 to 60% of maximal heart rate.	10–12 repetitions of seated row, pull-down, chest press, shoulder press, leg press, one leg lunge, dead bug and regular plank, with a weight adjusted individually.	52 weeks	No effect was found for hemodynamic variables after the intervention.
